# Nomogram for predicting major bleeding after off-pump coronary artery bypass grafting

**DOI:** 10.1186/s13019-024-02499-z

**Published:** 2024-01-23

**Authors:** Jianqin Zhu, Zhenjun Wu, Guiming Huang, Yuting Zhong, Cheng Peng

**Affiliations:** 1 Department of Anesthesiology, Ganzhou People’s Hospital, Ganzhou, 341000 China; 2 Department of Sleep Medicine, Ganzhou People’s Hospital, Ganzhou, 341000 China; 3 Department of Anesthesiology, The First Affiliated Hospital of Guangxi University of Chinese Medicine, Nanning, 530023 China; 4 Department of Anesthesiology, The First Affiliated Hospital of Gannan Medical University, Ganzhou, 341000 China

**Keywords:** Off-pump, Coronary artery bypass grafting, Bleeding, Nomogram

## Abstract

**Objective:**

The purpose of this investigation is to develop a novel nomogram for predicting major bleeding following off-pump coronary artery bypass grafting (CABG).

**Methods:**

Between January 2012 and December 2022, 541 patients who underwent off-pump isolated primary CABG were included in a retrospective analysis. The primary outcome measure after off-pump CABG was major bleeding. Based on the outcomes of a multivariate analysis, nomograms were constructed. Using receiver operating characteristic analysis and calibration, the predictive accuracy of the nomograms was assessed. Using decision curve analysis (DCA), the clinical benefit of the nomograms was determined.

**Results:**

We categorized 399 and 142 patients in the “no major bleeding group” and “major bleeding group”, respectively. Age (odds ratio (OR) 1.038; 95% confidence interval (CI) 1.009–1.068; *p* = 0.009), body mass index (OR 0.913; 95% CI 0.849–0.982; *p* = 0.014), hemoglobin (OR 0.958; 95% CI 0.945–0.971; *p* < 0.001), sodium (OR 0.873; 95% CI 0.807–0.945; *p* = 0.001), blood urea nitrogen (OR 1.198; 95% CI 1.073–1.338; *p* = 0.001), and operation time (OR 1.012; 95% CI 1.008–1.017; *p* < 0.001) were independent predictors for major bleeding after off-pump CABG. The model based on independent predictors exhibited excellent discrimination and calibration, with good agreement between actual and nomogram-estimated probabilities of generalization. DCA demonstrated that nomogram-assisted decisions have a greater positive benefit than treating all patients or none.

**Conclusions:**

The plotted nomogram accurately predicted major bleeding outcomes following off-pump CABG and may therefore contribute to clinical decision-making, patient treatment, and consultation services.

**Supplementary Information:**

The online version contains supplementary material available at 10.1186/s13019-024-02499-z.

## Introduction

Off-pump coronary artery bypass grafting (CABG) is a commonly used surgical procedure to treat coronary artery disease [1, 2]. It offers a number of advantages over conventional on-pump bypass surgery, including a decreased risk of neurocognitive dysfunction and systemic inflammatory response [3, 4]. However, off-pump CABG is not devoid of potential complications; major bleeding is a significant concern [5]. Major bleeding after off-pump CABG can result in an increase in morbidity, mortality, and hospital length of stay, posing a significant challenge to postoperative care and patient outcomes [6, 7]. Consequently, postoperative bleeding remains a significant concern in cardiac surgery.

Several studies have investigated the patient characteristics, comorbidities, procedural factors, and laboratory parameters that are associated with major bleeding after off-pump CABG [8, 9]. Existing prediction models, despite their extensive coverage and precision, have limitations. Constructing a prediction model specific to off-pump CABG that incorporates a vast array of relevant variables would tremendously aid clinicians in identifying patients at a higher risk of major bleeding and in determining the most effective interventions to prevent this complication.

A nomogram is a graphical instrument that allows for the prediction of individual risk by combining multiple risk factors into a single probability estimate [10]. As a prognostic instrument for a variety of tumor types, including endometrial stromal sarcoma, gastric cancer, and lung cancer, this method has been proposed as an alternative method or even a new standard method [11–13].

This study's objective is to develop a nomogram that integrates a variety of preoperative and intraoperative variables to predict the likelihood of major bleeding following off-pump CABG. This nomogram could be utilized as a valuable clinical tool for risk stratification, patient counseling, and optimizing perioperative care. Moreover, by identifying patients at high risk, proactive interventions can be implemented to reduce the incidence of severe bleeding and its associated negative outcomes.

## Materials and methods

### Study design

This single-center retrospective investigation was approved by the Ethical Committee of Ganzhou People's Hospital, and formal informed consent was waived. Between January 2012 and December 2022, data were collected from consecutive patients who underwent isolated primary off-pump CABG at Ganzhou People's Hospital by a single surgeon. Isolated primary off-pump CABG is the first coronary artery bypass graft surgery performed on a patient without cardiopulmonary bypass and without any other concomitant procedures. All of the anesthesiologists who participated in the study possessed a substantial understanding of cardiac anesthesia. Blood transfusion and fluid administration were regulated by anesthesiologists in accordance with an approved fluid management standard [14].

### Study population

The analysis screened for adult patients (≥ 18 years) who underwent isolated primary off-pump CABG and had comprehensive medical record information. Following are the exclusion criteria for this study: patients with emergency CABG; on-pump CABG; concomitant surgical procedures; patients with acute infection status and liver and kidney dysfunction as defined by the Goldman-Cecil Medicine Classification System [15]. 541 patients were selected after screening, and 5 patients were excluded due to incomplete information.

### Data collection

The following information was collected from patient medical records: age, gender, body mass index (BMI), smoking history, previous percutaneous transluminal coronary angioplasty (PTCA), previous surgery, underlying disease [hypertension, diabetes, pneumonia, cerebrovascular desease, myocardial infarction (MI), congestive heart-failure (CHF), and atrial fibrillation (AF)], and preoperative medicine use. left ventricular ejection fraction (LVEF) and. Laboratory indicators 24 h before surgery including white blood cell (WBC), platelet (PLT), platelet distribution width, platelet volume, plateletcrit (PCT), prothrombin time (PT), International normalization ratio (INR), hemoglobin, total protein, albumin, sodium, potassium, calcium, blood urea nitrogen (BUN), creatine, D-dimer, HDL cholesterol (HDL-C), LDL cholesterol (LDL-C), high sensitivity C reactive protein (HSCRP), Alanine transaminase (ALT), Aspartate transaminase (AST) were recorded. We also recoded intraoperative data [Multiarterial grafting, operation time, intraoperative heparin dosage, and Intraoperative protamine dosage]. The underlying disease were collected directly from the diagnosis at hospitalisation before the surgery. In the 4th Universal Definition of Myocardial Infarction (MI), myocardial injury differs from the term of MI. More specifically, the term MI should be used for myocardial injury with clinical evidence of acute myocardial ischaemia, plus the detection of a rise and/or fall in cTn values.3 Additionally, one of the following features has to be present: (1) symptoms of myocardial ischaemia; (2) new ischaemic electrocardiogram changes; (3) development of pathological Q waves; (4) imaging evidence of new loss of viable myocardium or new regional wall motion abnormality in a pattern consistent with an ischaemic aetiology; and (5) identification of a coronary thrombus by angiography or autopsy [16]. The diagnosis of AF is confirmed by history, physical examination, and electrocardiogram [17].

### Outcomes

The primary outcome of this investigation was major bleeding. According to the universal definition of perioperative bleeding (UDPB), the development of postoperative major bleeding was defined in classes 3 and 4. The UDPB defines five classes of perioperative bleeding to characterize the severity of bleeding, regardless of its source [18] (Additional file 1: Table S1).

### Statistical analysis

R 4.0.2 was used for statistical analysis (http://www.R-project.org). No missing data occurred for categorical variables. The loss of continuous variables was less than five percent, so mean values were substituted for the absent data. Continuous variables were reported as mean standard deviation (for normally distributed continuous variable) or median (interquartile ranges) (for non-normally distributed continuous variable). The Mann–Whitney U test or the student t test was used to compare the two groups. Categorical variables are presented as absolute numbers and percentages, and Pearson's chi-square test was used to compare the two groups. Using logistic regression models, the correlation between variables and major bleeding was determined. We then included age, sex, BMI, PCT, PT, hemoglobin, albumin, sodium, BUN, creatinine, D-dimer, ALT, operation time, and intraoperative protamine dosage into the stepwise regression model for analysis. Based on the results of the multivariable analysis and utilizing the rms package in R, a nomogram for evaluating major bleeding was developed. The calibration curve was drawn by repeated sampling 1000 times by Bootstrap method, with patients with major bleeding as the state variable and independent risk factors as the test variables. Analyses of receiver operating characteristic (ROC) curves were used to evaluate the performance of the established nomogram and other noninvasive markers for predicting major bleeding. Decision curve analysis (DCA) was used to evaluate the predictive performance in greater detail. A two-sided P value of less than 0.05 was considered statistically significant.

## Results

### Comparison of baseline characteristics

This study enrolled a total of 541 patients, with 399 individuals in the no major bleeding group and 142 patients in the major bleeding group. The age of the study patients was 64.80 ± 8.79 years old, and 415 (76.1%) were males. At baseline, patients who developed a major bleeding event were older, female, had lower BMI, PCT, hemoglobin, albumin, sodium, and ALT, had higher PT, BUN, D-dimer, creatine, operation time and intraoperative protamine dosage (Table 1).

**Table 1:** Baseline characteristics comparison between two groups of patients

Covariates	No major bleeding	Major bleeding	*p*
N	399	142	
Age (years)	63.91 ± 8.91	67.30 ± 7.97	< 0.001
Male, n (%)	317 (79.4)	98 (69.0)	0.016
BMI (kg/m^2^)	24.50 ± 3.07	23.48 ± 3.19	0.001
Smoking history, n (%)	147 (36.8)	45 (31.7)	0.317
Previous PTCA, n (%)	53 (13.3)	14 (9.9)	0.360
Previous surgery, n (%)	130 (32.6)	53 (37.3)	0.356
*Underlying disease*			
Hypertension, n (%)	260 (65.2)	98 (69.0)	0.466
Diabetes, n (%)	113 (28.3)	50 (35.2)	0.153
Pneumonia, n (%)	55 (13.8)	30 (21.1)	0.054
Cerebrovascular desease, n (%)	35 (8.8)	10 (7.0)	0.643
MI, n (%)	88 (22.1)	30 (21.1)	0.911
CHF, n (%)	63 (15.8)	32 (22.5)	0.092
AF, n (%)	20 (5.0)	4 (2.8)	0.393
LVEF (%)	0.62 ± 0.10	0.61 ± 0.10	0.830
*Preoperative medicine use*			
Statin, n (%)	383 (96.0)	136 (95.8)	1.000
LMWH, n (%)	381 (95.5)	136 (95.8)	1.000
Aspirin, n (%)	243 (60.9)	89 (62.7)	0.785
Clopidogrel, n (%)	195 (48.9)	66 (46.5)	0.695
β-blocker, n (%)	274 (68.7)	100 (70.4)	0.778
ACEI, n (%)	189 (47.4)	60 (42.3)	0.341
*Preoperative laboratory indicators*			
WBC, (× 10^12^/L)	6.40 (5.20, 7.70)	6.25 (5.30, 7.48)	0.409
PLT, (× 10^9^/L)	190.00 (163.00, 232.50)	188.00 (159.25, 224.00)	0.545
Platelet distribution width, (fL)	16.76 ± 1.01	16.75 ± 0.63	0.883
Platelet volume, (fL)	9.29 ± 1.18	9.22 ± 1.23	0.550
PCT, (%)	39.76 ± 4.60	36.38 ± 5.33	< 0.001
PT, (s)	11.47 ± 1.02	11.69 ± 1.02	0.032
INR	1.01 ± 0.88	1.02 ± 0.82	0.229
Fibrinogen, (mg/dL)	420.00 (350.50, 494.00)	430.00 (370.00, 520.00)	0.087
Hemoglobin, (g/dL)	13.35 ± 1.60	12.18 ± 1.80	< 0.001
Total protein, (g/dL)	6.69 ± 0.57	6.63 ± 0.63	0.246
Albumin, (g/dL)	3.99 ± 0.41	3.84 ± 0.42	< 0.001
Glucose, (mmol/L)	5.60 (4.93, 6.81)	5.36 (4.80, 6.43)	0.086
Sodium, (mmol/L)	140.93 ± 2.61	140.38 ± 3.02	0.037
Potassium, (mmol/L)	4.01 ± 0.33	4.02 ± 0.44	0.702
Creatinine, (mmol/L)	2.24 ± 0.12	2.23 ± 0.13	0.237
BUN, (mmol/L)	5.40 (4.45, 6.40)	5.90 (4.90, 7.40)	< 0.001
Creatinine, (μmol/L)	73.00 (63.00, 84.00)	80.00 (66.00, 100.50)	< 0.001
D-dimer, (µg/mL)	142.00 (82.50, 148.30)	148.30 (101.25, 202.75)	< 0.001
HDL-C, (mmol/L)	0.98 (0.87, 1.13)	1.00 (0.89, 1.21)	0.068
LDL-C, (mmol/L)	2.39 (1.91, 3.14)	2.40 (1.86, 3.04)	0.503
HSCP, (mg/L)	0.02 (0.01,0.05)	0.02 (0.01, 0.07)	0.061
ALT, (IU/L)	23.00 (17.00, 35.50)	20.00 (13.00, 31.00)	0.003
AST, (IU/L)	22.00 (18.00, 31.00)	22.00 (18.00, 33.00)	0.564
Operation time, (min)	281.00 (249.50, 320.00)	297.00 (265.00, 333.00)	< 0.001
Multiarterial grafting, n (%)	311 (77.9%)	117 (82.4%)	0.263
Intraoperative heparin dosage, (mg)	150.00 (110.00, 170.00)	150.00 (125.00, 200.00)	0.347
Intraoperative protamine dosage, (mg)	150.00 (100.00, 175.00)	150.00 (125.00, 200.00)	0.009
Cycle, n (%)			0.219
2012–2016	184 (46.1%)	74 (52.1%)	
2018–2022	215 (53.9%)	68 (47.8%)	

BMI: body mass index, PTCA: percutaneous transluminal coronary angioplasty, MI: myocardial infarction, CHF: congestive heart-failure, AF: atrial fibrillation, LVEF: left ventricular ejection fraction, LMWH: low molecular weight heparin, ACEI: angiotensin-converting enzyme inhibitors, WBC: white blood cell, PLT: platelet, PCT: plateletcrit, PT: prothrombin time, INR: International normalization ratio, BUN: blood urea nitrogen, HDL-C: HDL cholesterol, LDL-C: LDL cholesterol, HSCRP: high sensitivity C reactive protein, ALT: Alanine transaminase, AST: aspartate transaminase

### Identification of predictive factors for major bleeding

Using stepwise selection in the multiple logistic regression analysis model, six predictive variables [Age (odds ratio (OR), 1.038; 95% confidence interval (CI), 1.009–1.068; *p* = 0.009), BMI (OR 0.913; 95% CI 0.849–0.982; *p* = 0.014), hemoglobin (OR 0.958; 95% CI 0.945–0.971; *p* < 0.001), Sodium (OR 0.873; 95% CI 0.807–0.945; *p* = 0.001), BUN (OR 1.198; 95% CI 1.073–1.338; *p* = 0.001), Operation time (OR 1.012; 95% CI 1.008–1.017; *p* < 0.001)] were retained in the final simplified model and were used to construct the nomogram (Table 2).

**Table 2:** multiple logistic regression analysis

Covariates	β	SE	Wald	*p*	OR (95%CI)
Age (years)	0.038	0.014	7.067	0.009	1.038 (1.009–1.068)
BMI (kg/m^2^)	-0.091	0.037	5.999	0.014	0.913 (0.849–0.982)
Hemoglobin, (g/L)	-0.043	0.007	37.515	< 0.001	0.958 (0.945–0.971)
Sodium, (mmol/L)	-0.136	0.040	11.231	0.001	0.873 (0.807–0.945)
BUN, (mmol/L)	0.181	0.056	10.315	0.001	1.198 (1.073–1.338)
Operation time, (min)	0.012	0.002	28.371	< 0.001	1.012 (1.008–1.017)

BMI: body mass index, BUN: blood urea nitrogen

### Model development and validation

**Fig. 1:**
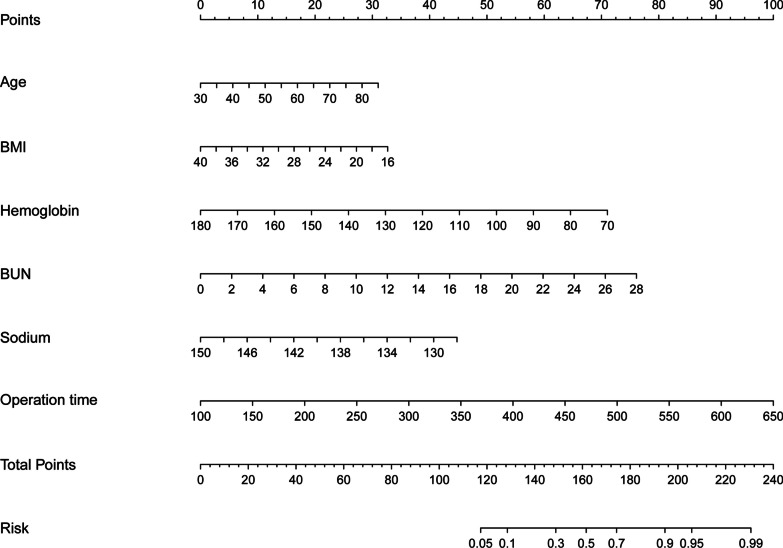
A constructed nomogram for predicting major bleeding after off-pump CABG

Figure 1 depicts the nomogram for predicting major bleeding probabilities. The nomogram is based on the proportional conversion of each regression coefficient from multivariable logistic analysis to a 0 to 100 point scale. Within the six variables that comprise the nomogram, each covariate was assigned a score by sketching a vertical line from the axis labeled points directly downward. Individual probabilities of major bleeding can be determined by summing the total score and locating it on the total points scale. Taking one participant as an example, the age was 65 years old and assigned 20 points, BMI was 28 kg/m^2^ and 17 points, HBG was 120 g/L and 39 points, BUN was 10 mmol/L and 27.5 points, Sodium was 130 mmol/L and 40 points, and the operation duration was 250 min and 27.5 points. The above total is 171 points, and the nomogram shows that the corresponding probability of major bleeding after surgery is about 0.7.

**Fig. 2:**
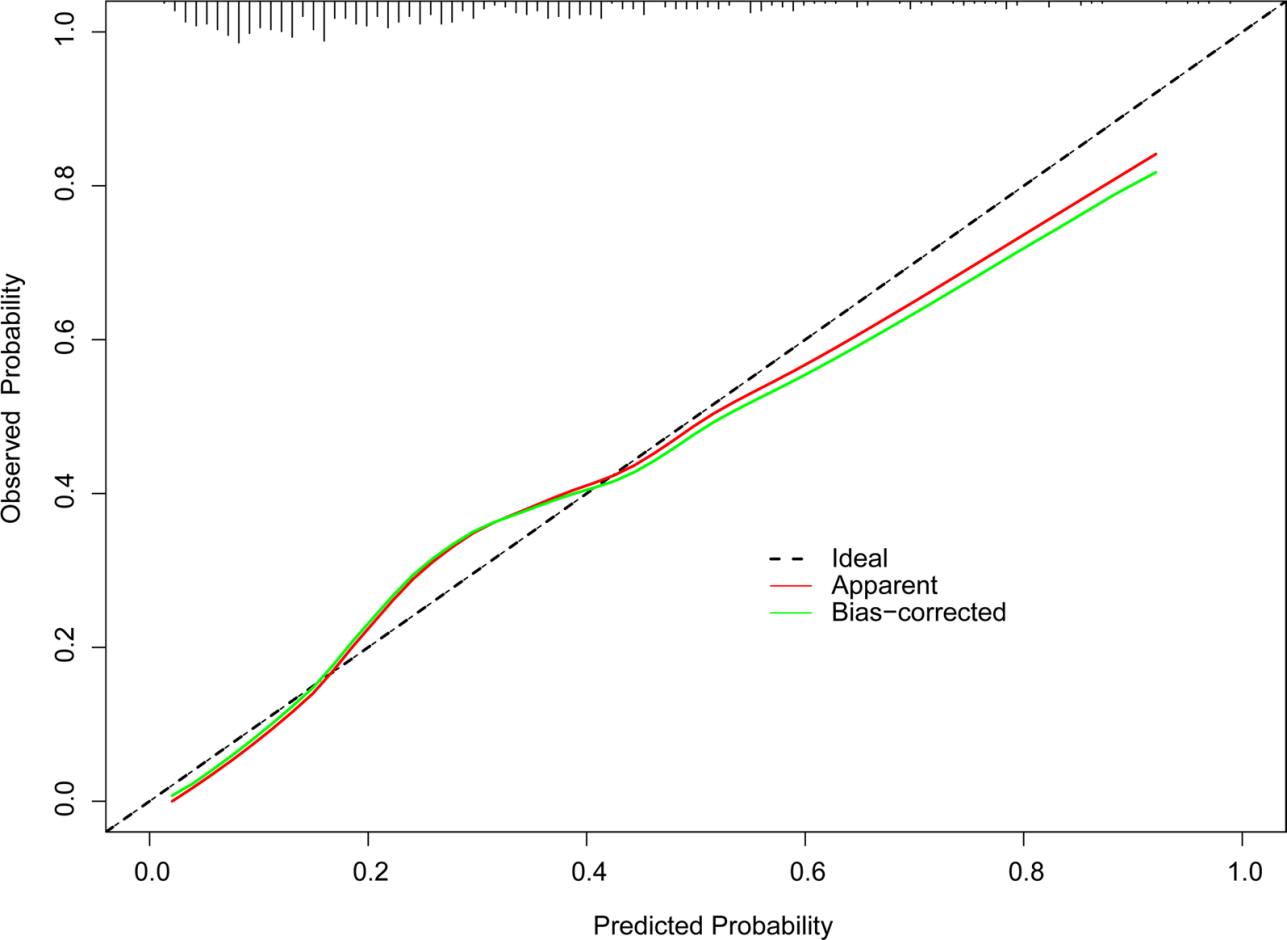
Calibration plots for estimating major bleeding probabilities

**Fig. 3:**
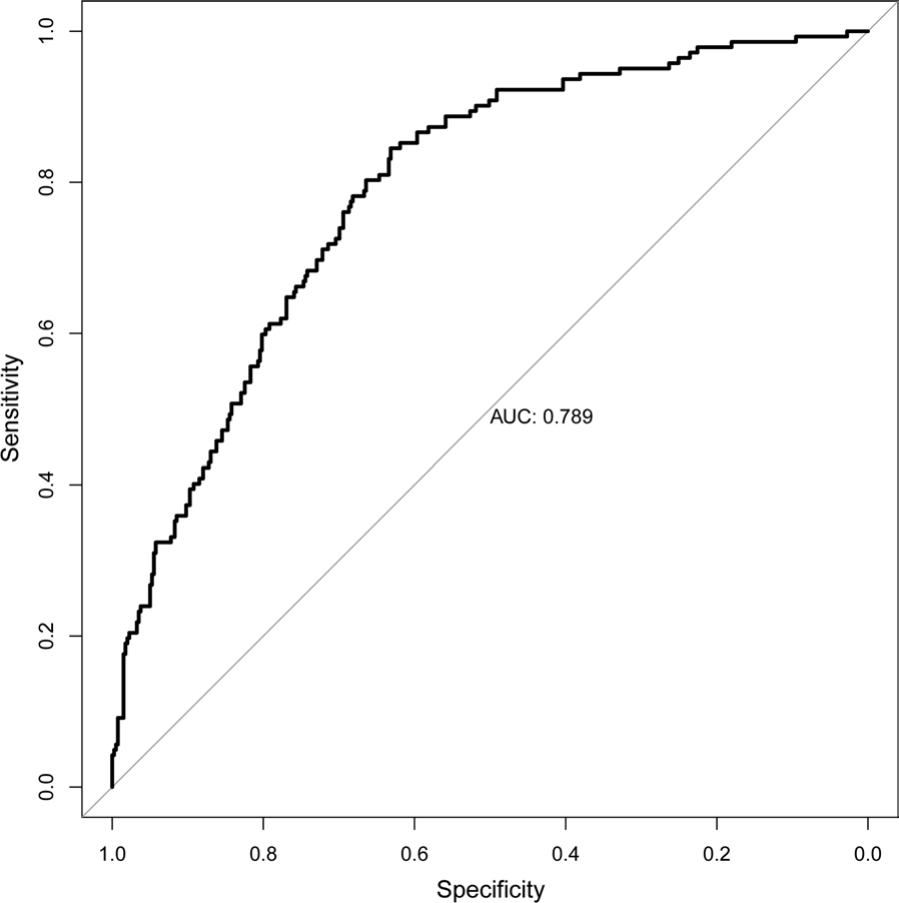
Areas under the curves of the model for predicting major bleeding probabilities

In addition, a calibration curve was produced to assess the efficacy of the nomogram. Calibration curves with 1,000 bootstrap resamples demonstrated reasonable agreement between the nomogram-predicted and actual generalization probabilities (Fig. 2: Mean absolute error = 0.026). The Hosmer–Lemeshow goodness of fit test indicates that this model is well-fitting (χ^2^ = 9.518, *p* = 0.301). Figure 3 depicts the ROC curves of the model predicting the probabilities of major bleeding. The area under the curve (AUC) of the model that predicted the probability of major bleeding was 0.789.

### Decision curve analysis

**Fig. 4:**
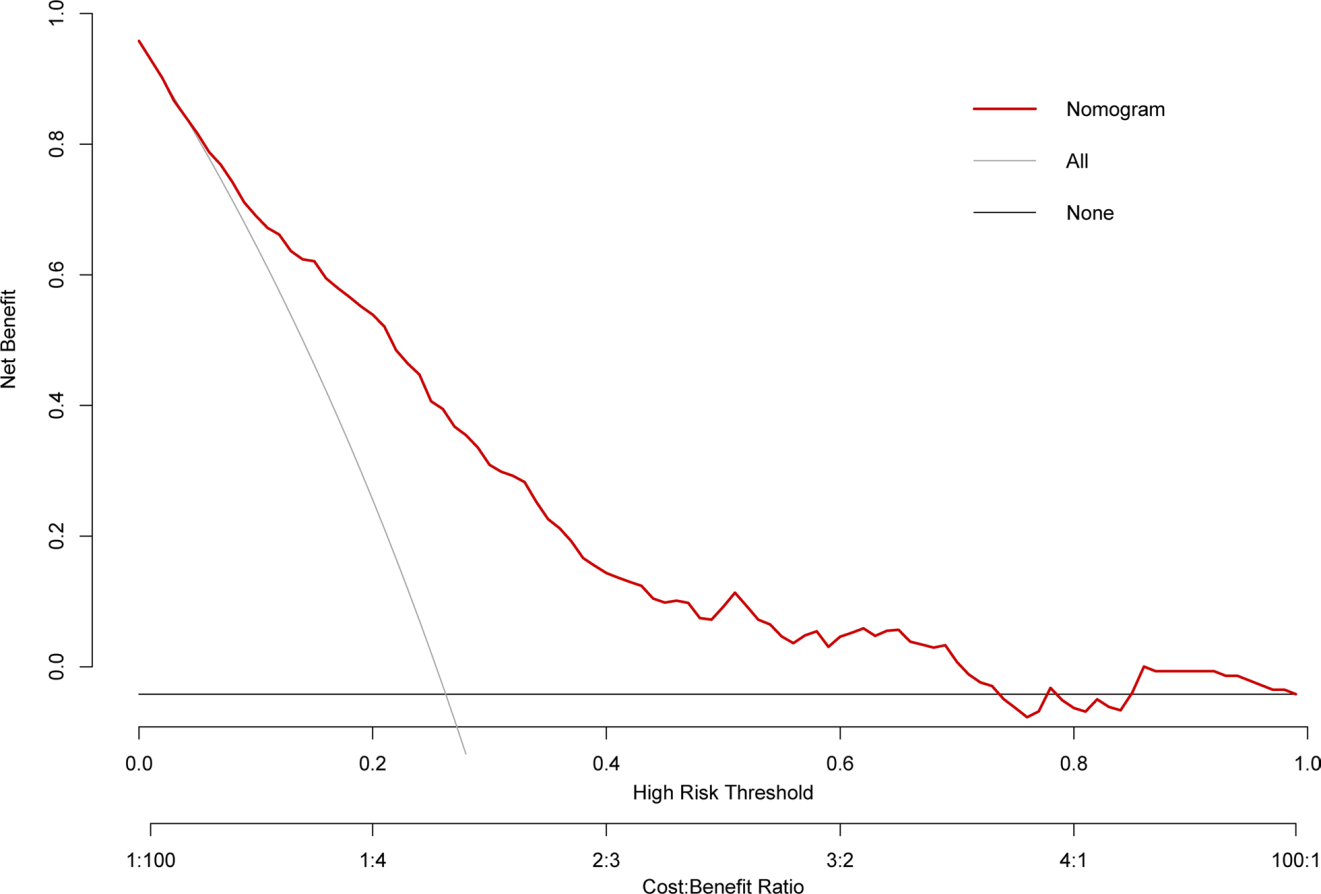
Decision curves of the nomogram model

Figure 4 demonstrates the net benefits of the nomogram model. Applying the model, the red line (corresponding to the model) has a significantly greater net benefit across a wide range of risk thresholds (8–75%) depicted in Fig. 4. In the nomogram model, the net benefit was 10% at a risk threshold of 60%. In other words, nomogram-assisted decisions to initiate treatment in high-risk patients provide a greater benefit than treating all patients or none.

## Discussion

Major bleeding is a major concern during off-pump CABG and can result in adverse outcomes and extended hospital stays. It is essential for risk stratification, appropriate patient counseling, and optimizing perioperative care to accurately predict the risk of severe bleeding. In this study, we devised a nomogram that combines preoperative and intraoperative variables to predict the likelihood of major bleeding following off-pump CABG. The nomogram demonstrated excellent discrimination, calibration, and clinical utility, providing clinicians with a valuable instrument for making informed decisions about bleeding risk and implementing preventative measures.

The nomogram was derived from a retrospective analysis of 541 patients who underwent off-pump isolated primary CABG from January 2012 to December 2022. Age, BMI, hemoglobin, sodium, BUN, and operation time were identified as independent predictors of major bleeding. These predictors have been previously associated with hemorrhage complications in cardiac surgery and are consistent with previous research. Consistently, older age has been identified as a risk factor for hemorrhage due to factors including decreased vascular integrity and impaired hemostatic function [19]. Careful preoperative assessment, closer monitoring, and tailored interventions for older patients may help mitigate the risk. Low BMI has been associated with an increased risk of bleeding, possibly owing to lower coagulation factor levels [20]. Nutritional optimization and targeted interventions to address potential bleeding risks in patients with lower BMI may be beneficial. Hemoglobin and sodium levels reflect the overall health status of the patient and can affect hemostasis [21]. Preoperative correction of anemia and maintaining electrolyte balance during and after surgery can be crucial in preventing major bleeding. Renal dysfunction, which can compromise platelet function and coagulation, may be indicated by elevated BUN levels [22, 23]. Therefore, preoperative assessment and management of renal function, along with adequate hydration, may be essential in reducing bleeding risk. Extended operation Time is a recognized risk factor for bleeding because it subjects the patient to protracted anticoagulation and surgical trauma [24]. Strategies to optimize surgical efficiency, meticulous hemostasis, and consideration of minimally invasive techniques may help reduce operation time and subsequently lower the risk of major bleeding.

Our developed nomogram demonstrated good discrimination, with an AUC of 0.789, indicating its ability to differentiate between patients who experienced major bleeding and those who did not. The calibration curves revealed excellent concordance between the predicted probabilities and the observed probabilities, indicating that the nomogram was properly calibrated. These results suggest that the nomogram provides accurate and dependable estimates of an individual's risk of hemorrhage after CABG without a pump.

In addition to discrimination and calibration, DCA was used to evaluate the clinical utility of the nomogram. DCA permits the assessment of the net benefit of using a predictive model in clinical decision-making. The DCA demonstrated that the nomogram-assisted decisions had a greater net benefit across a broad range of risk thresholds than treating all patients or none. Incorporating the nomogram into clinical practice can enhance patient outcomes by identifying high-risk patients who would benefit from targeted preventative measures.

Nomograms are gaining popularity in clinical practice due to their ability to provide individualized risk estimations based on multiple variables. Nomograms have advantages over conventional prediction models because they provide a visual representation of risk probability, facilitate risk communication, and aid in decision-making. They have been extensively utilized for diagnosis and treatment planning in a variety of medical specialties and tumor types. Our study extends the application of nomograms to the prediction of major bleeding after off-pump CABG, thereby providing clinicians with a valuable tool for individualizing patient care.

It is essential to recognize the limitations of our investigation. First, it was a single-center retrospective study, which may have introduced selection bias and restricted the generalizability of the results. There is a need for additional validation studies in larger and more diverse patient populations to confirm the performance of the nomogram. Secondly, the variables included in the nomogram were derived from data available in our institution's database, and there may be other factors that influence hemorrhage risk but were not accounted for in our analysis. Future research could investigate the incorporation of additional pertinent variables to enhance the nomogram's predictive accuracy. Even though the nomogram demonstrated excellent discrimination and calibration, prospective studies are required to determine the efficacy of using the nomogram to guide preventive interventions and improve patient outcomes.

We developed a nomogram to predict major bleeding after off-pump CABG. Incorporating preoperative and intraoperative variables, the nomogram demonstrated excellent discrimination, calibration, and clinical utility. The nomogram can serve as a valuable clinical decision-making instrument, assisting clinicians in identifying patients at increased risk of major bleeding and directing the implementation of preventive measures. Interventions can be tailored to reduce bleeding complications and enhance patient outcomes in off-pump CABG procedures by proactively identifying high-risk patients. Further prospective validation and implementation studies are required to determine the nomogram's broad applicability and impact on patient care.

### Supplementary Information

**Additional file 1: Table S1**. Bleeding categories according to the UDPB in adult cardiac surgery (if different categories indicate mixed definitions of bleeding, the worst definition applies)

## Data Availability

The datasets used and analysed during the current study are available from the corresponding author on reasonable request.

## Abbreviations

ACEI: Angiotensin-converting enzyme inhibitors
ALT: Alanine transaminase
AF: Atrial fibrillation
AST: Aspartate transaminase
AUC: Area under the curve
BMI: Body mass index
BUN: Blood urea nitrogen
CABG: Coronary artery bypass grafting
CHF: Congestive heart-failure
CI: Confidence interval
DCA: Decision curve analysis
HDL-C: HDL cholesterol
HSCRP: High sensitivity C reactive protein
INR: International normalization ratio
LDL-C: LDL cholesterol
LMWH: Low molecular weight heparin
LVEF: Left ventricular ejection fract
MI: Myocardial infarction
OR: Odds ratio
PCT: Plateletcrit
PLT: Platelet
PT: Prothrombin time
PTCA: Percutaneous transluminal coronary angioplasty
ROC: Receiver operating characteristic
UDPB: Universal definition of perioperative bleeding
WBC: White blood cell
