# Supplementary material for: Nomogram for predicting major bleeding after off-pump coronary artery bypass grafting
Source: J Cardiothorac Surg. 2024 Jan 23;19:23. doi: 10.1186/s13019-024-02499-z (PMC10807166; doi:10.1186/s13019-024-02499-z)
Supplement: Supplementary file 1 — Additional file 1: Table S1. Bleeding categories according to the UDPB in adult cardiac surgery (if different categories indicate mixed definitions of bleeding, the worst definition applies) [file 13019_2024_2499_MOESM1_ESM.docx]

Table S1 Bleeding categories according to the UDPB in adult cardiac surgery (if different categories indicate mixed definitions of bleeding, the worst definition applies)

| **Bleeding definition** | **Sternal closure delayed** | **Postoperative chest tube blood loss within 12 hours (mL)** | **PRBC (units)** | **FFP (units)** | **PLT (units)** | **Cryoprecipitate** | **PCCs** | **rFVIIa** | **Reexploration**  **/tamponade** |
| --- | --- | --- | --- | --- | --- | --- | --- | --- | --- |
| Class 0 (insignificant) | No | <600 | 0 | 0 | 0 | No | No | No | No |
| Class 1 (mild) | No | 601-800 | 1 | 0 | 0 | No | No | No | No |
| Class 2 (moderate) | No | 801-1000 | 2-4 | 2-4 | Yes | Yes | Yes | No | No |
| Class 3 (severe) | Yes | 1001-2000 | 5-10 | 5-10 | N/A | N/A | N/A | No | Yes |
| Class 4 (massive) | N/A | >2000 | >10 | >10 | N/A | N/A | N/A | Yes | N/A |

UDPB, Universal definition for perioperative bleeding; PRBC, packed red blood cells; FFP, fresh frozen plasma; PLT, platelet concentrates; PCCs, prothrombin complex concentrates; rFVIIa, recombinant activated factor VII; N/A, not applicable.
